# The Predictive Role of Biomarkers and Genetics in Childhood Asthma Exacerbations

**DOI:** 10.3390/ijms22094651

**Published:** 2021-04-28

**Authors:** Emanuela di Palmo, Erika Cantarelli, Arianna Catelli, Giampaolo Ricci, Marcella Gallucci, Angela Miniaci, Andrea Pession

**Affiliations:** 1 Pediatric Unit–IRCCS Azienda Ospedaliero-Universitaria di Bologna, 40138 Bologna, Italy; emanuela.dipalmo@aosp.bo.it (E.d.P.); marcella.gallucci@aosp.bo.it (M.G.); angela.miniaci@aosp.bo.it (A.M.); 2 Specialty School of Paediatrics-Alma Mater Studiorum, Università di Bologna, 40138 Bologna, Italy; erika.cantarelli@studio.unibo.it (E.C.); arianna.catelli@studio.unibo.it (A.C.); 3 Medical and Surgical Sciences (DIMEC), University of Bologna, 40138 Bologna, Italy; giampaolo.ricci@unibo.it

**Keywords:** asthma, exacerbation, exacerbation risk, exacerbation prevention, prevention, biomarkers, non-invasive, genetic

## Abstract

Asthma exacerbations are associated with significant childhood morbidity and mortality. Recurrent asthma attacks contribute to progressive loss of lung function and can sometimes be fatal or near-fatal, even in mild asthma. Exacerbation prevention becomes a primary target in the management of all asthmatic patients. Our work reviews current advances on exacerbation predictive factors, focusing on the role of non-invasive biomarkers and genetics in order to identify subjects at higher risk of asthma attacks. Easy-to-perform tests are necessary in children; therefore, interest has increased on samples like exhaled breath condensate, urine and saliva. The variability of biomarker levels suggests the use of seriate measurements and composite markers. Genetic predisposition to childhood asthma onset has been largely investigated. Recent studies highlighted the influence of single nucleotide polymorphisms even on exacerbation susceptibility, through involvement of both intrinsic mechanisms and gene-environment interaction. The role of molecular and genetic aspects in exacerbation prediction supports an individual-shaped approach, in which follow-up planning and therapy optimization take into account not only the severity degree, but also the risk of recurrent exacerbations. Further efforts should be made to improve and validate the application of biomarkers and genomics in clinical settings.

## 1. Introduction

**Figure 1:**
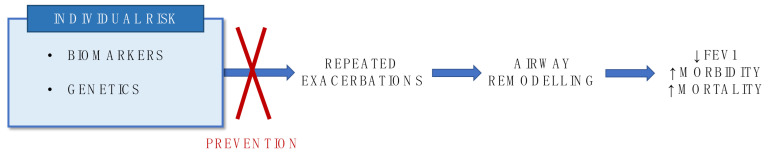
Biomarkers and genetics as useful tools to identify asthma exacerbators at risk of lung function impairment.

Asthma is the most common chronic disease in childhood. WHO surveillance highlighted a rising trend of global asthma prevalence [1]. In particular, asthma exacerbations are relevant causes of children morbidity and mortality. Acute care visits, emergency room treatments and hospitalization account for the major proportion of asthma-related economic expenditure [2]. Global Initiative for Asthma (GINA) defines exacerbations as episodes characterized by a progressive increase in symptoms of shortness of breath, cough, wheezing or chest tightness and progressive decrease in lung function, representing a change from the patient’s usual status sufficient to require a change in treatment [3]. These features are the result of chronic inflammation of the lower airways, which predisposes one to airway hyperresponsiveness and obstruction. A cellular infiltrate, including eosinophils and other cell types (neutrophils, monocytes, lymphocytes, mast cells), contributes to airflow restriction, both filling peripheral airways and releasing inflammatory mediators responsible for epithelial desquamation and increased smooth muscle contractility [3]. Asthma exacerbations result from the complex interaction between individual susceptibility factors, including genetic predisposition and immune response, and environmental triggers like infections, allergens and atmospheric pollution [4]. Genetic variants and host immune system can predispose to an increased responsiveness towards environmental exposures. Common respiratory viruses are the main cause of exacerbations in both children and adults [5,6]. Particularly, rhinoviruses are the most frequently associated agents, being involved up to 80% of asthma attacks, especially in seasonal peaks [6]. Allergen exposure, besides representing a major trigger itself, interacts with viruses, increasing exacerbation risk in childhood [7]. Similarly, indoor allergens like dust mite can initiate airway inflammation predisposing one to hyperreactive responses to other irritants. A further significant trigger is thunderstorm weather; rainfall and humidity induce osmotic shock rupture of aeroallergens releasing smaller respirable particles, and concentrate allergen load in a band of air at ground level [8,9]. In addition, urbanization enhanced the contribution of outdoor pollution to asthma exacerbation rate, especially evident in high-income countries [10]. Many studies have highlighted that recurrent asthma attacks significantly contribute to progressive loss of lung function, as shown by excessive decline in FEV1. The worsening of airway inflammation during recurrent exacerbations induces permanent structural changes, like basement membrane thickening, smooth muscle hypertrophy, vascular changes, and mucus hypersecretion. The airway remodeling results in increased disease severity. Asthma exacerbation can be fatal or near-fatal (requiring mechanical ventilation), even in case of apparently mild asthma [11]. Many patients with mild disease are not diagnosed and indeed do not receive any treatment, or the therapy is not consistent with guidelines. Compliance is also poor in these subjects, due to underestimation of symptoms [12]. Although exacerbations are less frequent in patients with mild-moderate than those with severe asthma (uncontrolled despite maximal optimized therapy [13]), a recent systematic review stated that up to 22% of these patients required hospitalization or had a severe exacerbation in the previous year. Thus, mild asthma represents a new burden, including a considerable risk of life-threatening events [14]. These findings support the relevance of exacerbation prevention as primary target in asthma management for all patients. In recent years, efforts focused on the identification of subjects ‘exacerbation-prone’ with higher risk of recurrent asthma attacks (Figure 1) [15,16]. Our work aims to review current advances in the identification of exacerbation predictive factors, focusing on the role of non-invasive biomarkers and genetics in childhood.

## 2. Methods

We conducted research on PubMed database selecting the most relevant articles published between 2005 and 1 February 2021. We used the search terms “asthma” and “exacerbation” and “biomarkers” or “genetic”. A restriction for English language was applied. Articles were examined by title, abstract, or full text. We included all randomized clinical trials, retrospective studies, meta-analysis, systematic reviews, and literature reviews published. In addition, the reference lists of all selected articles were evaluated (flowchart in Figure S1).

## 3. Biomarkers

A biomarker is a defined characteristic that is measured as an indicator of normal biological processes, pathogenic processes, or biological responses to an exposure or intervention, including therapeutic interventions. Particularly, a predictive biomarker is a marker used to identify individuals who are more likely than similar individuals without the biomarker to experience a favorable or unfavorable effect from exposure to a medical product or an environmental agent [17]. In asthma, the role of biomarkers has been largely studied in diagnosis, prediction of asthma severity, prognosis and treatment response. About exacerbation, their value is not well defined yet. Biomarkers may be useful in assessing and studying the biology of exacerbation, but a more precious function may be recognizing patients at increased risk for exacerbations. In children a major challenge is the identification of biomarkers in easier-to-collect sample, because of the limits in sample yield (e.g., sputum) in younger subjects (Table 1).

### 3.1. Eosinophils

**Figure 2:**
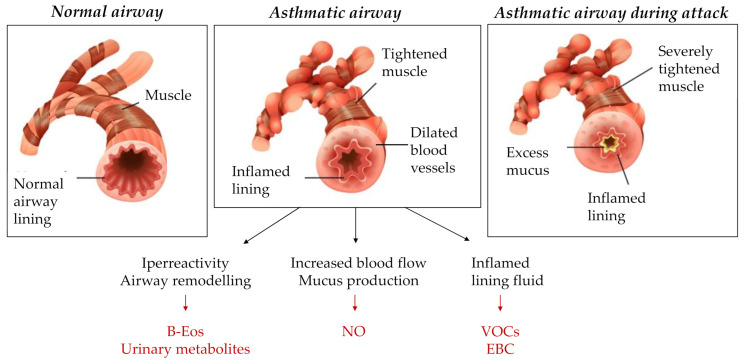
Biomarkers as indicators of pathological modification in asthmatic airways. Abbreviations: B-Eos = blood eosinophils; NO = nitric oxide; VOCs = volatile organic compounds; EBC = exhaled breath condensate. Image in collaboration with: Freepik.com.

Eosinophils are the central driver of type 2 (T2) inflammation and play a crucial role in maintaining airway inflammation of patients that usually respond well to inhaled corticosteroids [39]. These leucocytes are the predominant inflammatory cells in asthmatic airways and contribute to the pathophysiology of exacerbations, through the interactions with adaptive immune cells and the release of toxic granules involved in airway hyper-responsiveness and bronchoconstriction. Eosinophils influence chronic airway modifications, through mediators as transforming growth factor-β (TGF-β) implicated in remodeling pathway [40] (Figure 2). The clinical relevance of these cells in asthma has been supported by the observation of more frequent exacerbations in patients with sputum eosinophil counts higher than 3% [19]. In adults eosinophils in induced sputum samples have been used in many trials as markers of T2 inflammation. This procedure is not feasible in younger children and even in preadolescents it is burdened with execution difficulties (i.e., sample yield) [41]. Non-invasive markers of T2 inflammation are needed to more easily identify exacerbation-prone asthma patients that require closer follow-up and therapy optimization [23]. Peripheric blood eosinophils (B-Eos) have been largely tested as an alternative exam. B-Eos have a lower specificity than sputum eosinophils because of the influence of potential confounding factors, such as allergen exposure, parasitic infections, and current corticosteroid therapy. However, B-Eos have been shown to be significantly predictive of sputum eosinophilia [21]. In adults, numerous works observed the association between elevated B-Eos and asthma attacks, with increasing rate of hospitalization [24,42,43]. In childhood, the role of blood eosinophils in the prediction of asthma exacerbations has long been unclear. Some studies stated the validity of this association in children and adolescents [18,19,22,24]; however, the cut-point of blood eosinophils for clinical detection of type 2 inflammation was not precisely determined. A retrospective study of children 5 to 11 years in a large medical care organization compared different B-Eos thresholds and identified an optimal cut-off of 300 cells/mL, which was associated with a 52% increase in asthma exacerbation rate [21]. The same limit value was utilized by other authors [19,20,24]. Interestingly, Pavord et al. in DREAM trial studied 621 patients aged 12–74 years and considered B-Eos count > 300 cells/mL as a biomarker to identify subjects with recurrent asthma exacerbations eligible to Mepolizumab therapy. The treatment reduced clinically significant exacerbations with concomitant lower blood and sputum eosinophil levels, supporting the predictive value of B-Eos [44]. A recent analysis, focusing on infant and adolescent populations, reported differences by age group in optimal B-Eos cut-points, being lower for children 5 to 11 years (>150 cells/mL) than for adolescents (>300 cells/mL). Exacerbation risk was more than two-folds raised [23]. Different findings in previous cited cut-points could be due to sociodemographic variability among the studied populations. This association was not confirmed in all studies in adults [15,45]. A recent observation regards the fluctuation trend of biomarkers, supporting the use of multiple measurements, rather than single-point assessment, and of composite biomarkers to better identify exacerbation-prone asthma [46]. The predictive value of blood eosinophils has yet to be tested in a prospective study in childhood.

### 3.2. Fractional Exhaled Nitric Oxide

Fractional exhaled nitric oxide (FeNO) is a relevant mediator associated with eosinophilic airway inflammation in asthmatic patients. Nitric oxide (NO) regulates pulmonary blood flow, mucus production, ciliary activity and inflammation (Figure 2). Physiologically, NO is produced at low levels by constitutive form of NO synthase (NOS). In cases of airway inflammation pro-inflammatory cytokines upregulate inducible NOS, producing high concentrations of NO. In the context of asthma, this inflammatory response results in worsening of airway obstructive symptoms [47]. The role of FeNO as biomarker has been largely investigated in the adult population, while in childhood its clinical value is incompletely understood. FeNO levels were described in relation with increased exacerbation rates (up to 2.4–fold) [20,25,47]. Not all studies on children confirmed the association [23,43]. Similar to that previously discussed about eosinophils, a limited utility of a single FeNO measurement was reported, due to the fluctuation of FeNO levels [48]. Analogous results were achieved combining FeNO concentrations and clinical characteristics [49]. The analysis of the variation in daily FeNO measurements and cross-correlation with symptom scores allowed to identify patients who worsened. However, this approach has limited availability [50]. A significant reduction of exacerbation risk occurred performing a FeNO-guided asthma therapy in systematic review [21]. A recent metanalysis showed that modifying asthma management according to FeNO levels reduces the numbers of children with asthma exacerbations and their frequency (RR 0.73) [51]. However, nitric oxide levels did not prove to impact on the day-to-day symptoms [52]. Furthermore, the benefits of FeNO-based algorithms have been observed to occur at the expense of increased treatment burden [53,54]. Adult trials were recently performed in order to define the FeNO cut-point predictive of acute attacks [45,55]. This field is poorly investigated in childhood. Zeiger et al. conducted a multicenter cross-sectional retrospective study of atopic 12- to 56-year-old persistent asthmatics. They reported that highest (≥48 ppb) versus lowest quartile (≤19 ppb) FeNO levels were associated with excess short-acting b2-agonist use and exacerbations requiring oral corticosteroid courses in the previous year, independent of asthma control tool and spirometry (RR 2.40) [27]. The cut-off of 20 ppb was proposed even in the randomized control trials with FeNO-guided therapeutic approach of Peirsman [53] and Petsky [54]. Further studies in children and adolescents are needed in order to obtain a validated cut-off and a more practical interpretation of FeNO. The combination of high FeNO levels with elevated blood eosinophils was associated with increased morbidity including exacerbations, with a reported 2-fold increased risk [56,57]. This supports the use of composite biomarkers.

### 3.3. Volatile Organic Compounds

Exhaled breath condensate (EBC) is a non-invasive test which measures airway inflammation by the collection of airway secretions [31]. The condensate contains unstable volatile (H2O2) and semi- and non-volatile molecules (proteins and cytokines) carried by respiratory droplets. Its composition mirrors that of the airway lining fluid (Figure 2), enabling a non-invasive study of pulmonary inflammatory state [58]. Exhaled volatile organic compounds (VOCs) function as marker of airway inflammation, originating from the reaction between reactive oxygen species and cell membranes. VOCs showed a predictive power for asthma attacks [28]. More recently, Vliet et al. used a profile of 15 VOCs in a first study. Children with persistently controlled asthma were well distinguished from children with persistently uncontrolled disease [29]. In a subsequent one-year prospective observational study on 96 asthmatic children, they identified 7 VOCs in exhaled breath showing to be predictive of asthma exacerbations in children within 14 days after sampling (3 aldehydes, 1 hydrocarbon, 1 ketone, 1 aromatic compound, and 1 unidentified VOC). This model presented a sensitivity of 88% in predicting exacerbations within 14 days, and 63% within 21 days [30].

### 3.4. Other Molecules Of Exhaled Breath Condensate

A prospective study on a small sample found interleukin (IL)-5 of EBC having a significant predictivity of asthma exacerbation. IL-5 is a glycoprotein produced by Th-2 cells and mast cells, that induces activation of eosinophils promoting airway inflammation [32]. Regarding eicosanoids in EBC, earlier literature reported mixed results. 8-isoprostane (8-IP) showed increased levels in childhood asthma, and 8-IP levels in this study were higher in children having >4 exacerbations per year compared to children with 1–4 exacerbations per year [31]. Otherwise, Vliet et al. examined EBC every 2 months for 12 months, and the predictive power of acidity and cytokines was rather poor [49]. A major issue continues to be the lack of standardization regarding EBC collection, preservation and analysis. This results in heterogeneous and conflicting data, questioning the validity of findings [59]. The potential role of EBC needs multicenter studies to achieve standardization.

### 3.5. IL-6

Blood IL-6 has been recently identified as an interesting biomarker of asthma in adults. Jevnikar et al. described a specific gene expression profile induced by IL-6 trans-signaling (TS) distinct from the T2 inflammation gene signature. A novel asthmatic patient subset was defined, with IL-6TS pathway acting as biological driver in lung epithelium. These patients were characterized by non-T2 airway inflammation and increased exacerbation rate with poor asthma control [60]. A longitudinal analysis on a population of exacerbation-prone asthmatics was performed in adults. High plasma IL-6 was observed, and the incident rate ratio for exacerbation augmented by 10% for each 1 pg/uL increase in baseline IL-6 level [15]. The role of IL-6 has been poorly studied in childhood. Li et al. studied the association of higher IL-6 levels and asthma hospitalizations and systemic corticosteroid use in a population of 12 years and older subjects [34]. The recent work of Jackson, et al. investigated the potentiality of this biomarker in 717 participants aged 6 to 17 years. A significant association was observed between baseline IL-6 level and the probability of experiencing an asthma attack treated with systemic corticosteroids during the 1-year study. The risk increased by 24% for each quartile elevation in serum IL-6 level. Unlike previous studies in adults, in children IL-6 showed lower blood levels, rarely above 3.1 pg/mL, and did not correlate with asthma severity. An interesting speculation is that children presenting frequent exacerbations and lower IL-6 levels may become adults with severe asthma phenotype [33].

### 3.6. Urinary Metabolites

Urine is a body fluid of growing attraction in the latest years, because it is easy to collect even in younger children and rich in metabolites. Urinary Bromotyrosine (BrTyr) has been studied as potential biomarker of eosinophilic asthma. Activated eosinophils secrete granule proteins, including an eosinophil peroxidase involved in pathway leading to production of 3-bromotyrosine (Figure 2). The latter can be measured in urinary samples and represents a marker of eosinophil activation [61,62]. Furthermore, the distinction between eosinophil levels, as determined by blood or sputum eosinophil counts, and eosinophil activation, as determined by BrTyr levels, support the observation that BrTyr levels closely mirror asthma symptoms and risk of exacerbation than eosinophil count. Wedes, et al. observed that high levels of BrTyr conferred 4.0-fold the odds of having an asthma exacerbation in the subsequent 6 weeks [35]. Urinary leukotriene E4 (uLTE4) is a biomarker of total body cysteinyl leukotriene (CysLT). High uLTE4 levels may represent an inflammatory condition. In asthma pathogenesis, CysLT have bronchoconstrictive activity and contribute to increasing the proliferation and recruitment of eosinophils. Urinary LTE4 levels are influenced by asthma triggers, such as air pollution, tobacco smoke exposure, and upper respiratory infections, explaining the role of CysLTs as mediators of asthma exacerbation [36]. Rabinovitch and colleagues reported that high uLTE4 levels (cut-off 106 pg/mg measured by mass spectrometry assay) produced a 100% positive predictive value and a 78% negative predictive value for risk of emergency department or urgent care visits in children exposed to secondhand tobacco smoke [63]. A recent clinical observational trial in adult and adolescent populations examined urinary metabolites of prostaglandins (PGs), cysteinyl leukotrienes, and isoprostanes. High concentrations of LTE4 and PGD2 metabolites, biomarkers of T2 inflammation, correlated with lower lung function and increased FeNO and eosinophil count in blood, sputum and urine. Furthermore, these patients received systemic corticosteroids more frequently. High isoprostane concentrations, marker of non-T2 inflammation, were associated with more frequent exacerbations [37].

### 3.7. Salivary Biomarkers

Saliva is another promising body fluid for disease diagnosis and prognosis especially in children because it is noninvasive, simple and low-cost. Specific cytokine profiles in saliva have been associated with asthmatic phenotype [64,65]. A strong correlation between symptom control and salivary levels of eotaxin, IL-5 and IL-8 was reported in both children and adults with asthma [38]. Another study assessed salivary surfactant protein D (SP-D) levels [66], known to be secreted from the salivary glands, as well as from type II alveolar epithelial cells and nonciliated Clara cells [67]. They observed that elevated SP-D may reflect asthmatic inflammation in peripheral small airways and the risk of losing asthma control. Authors indeed found a correlation of SP-D levels with both the increased resistance of peripheral airway measured with forced oscillation technique and the clinical severity of asthma exacerbations [66]. A recent comparative proteomic analysis between healthy and asthmatic groups, despite performed on small population sample, identified significant modifications in salivary proteins using gel-based quantitative proteomics. This study found 6 proteins in salivary sample related to uncontrolled asthma: nucleoside diphosphate kinase, fibrocystin, zinc finger protein 263, uncharacterized LOC101060047 (ENSG00000268865), desmoglein 2 and S100A2. Nucleoside diphosphate kinases plays a role in mucus production pathway. Fibrocystin seems to regulate mucocilliary sensing and transport within pulmonary airways. Desmoglein-2 functions in cellular adhesion and epithelial barrier. Little is known about function of the other proteins in asthma [68]. These findings should support further research on the potential predictive role of salivary proteomics, even as exacerbation biomarkers.

## 4. Genetic and Transcriptomic Predisposition 

**Figure 3:**
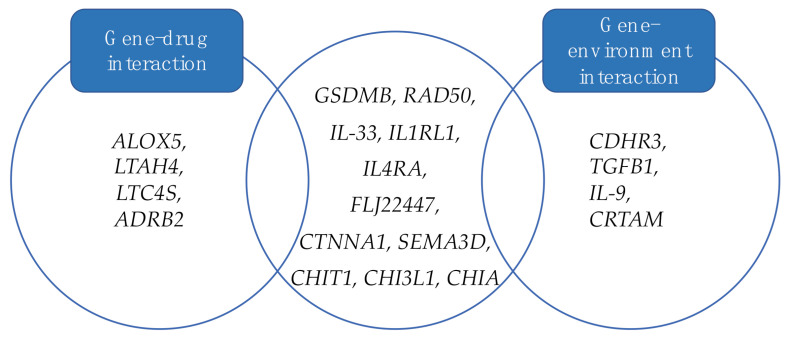
Genes implicated in asthma exacerbation pathogenesis, including gene-drug and gene-environment interactions.

The role of heritability in the complex pathogenesis of asthma is higher for childhood-onset asthma than adult asthma, although a genetic overlap between these two populations exists [69]. Identification of asthma susceptibility genes has grown rapidly over the last years, especially with the application of the genome-wide association study (GWAS) and pooled GWAS approach [70]. More recent analyses have concentrated on the role of genetic predisposition in asthma exacerbation. Given the large heterogeneity of asthma pathogenesis, a different strategy starting from specific phenotypes observation, that are more likely to be driven by genetic mechanisms, might help to increase the power of genetic studies [71]. In some cases, an overlap between previously reported asthma susceptibility genes and exacerbation-predictive single nucleotide polymorphisms (SNPs) has been identified, as multiple common variants in the same genes could contribute both to disease onset and asthma attacks (Table 2). Genetic predisposition to asthma exacerbation involves genes implicated in both intrinsic asthma pathogenesis and gene-environment interaction [4] (Figure 3). In the last years, there has been growing interest on epigenetic mechanisms in childhood asthma. Studies investigated their correlation with specific features of asthma, like severity or drug sensitivity [72]. Alongside the role in understanding the clinical heterogeneity of asthma, epigenetics may be a useful tool for exacerbation prediction.

### 4.1. GSDMB

Gasdermin B (*GSDMB*) locus on chromosome 17q21 is associated with childhood-onset asthma in ethnically different populations [101] In close proximity to *GSDMB*, *ORMDL3* gene showed a highly reproducible association with childhood onset asthma at GWAS [101,102]. *ORMDL3* encodes a protein of endoplasmic reticulum involved in different downstream pathways including calcium channel signaling, sphingolipids synthesis, unfolded-protein response, and it has also been associated with inflammation [103]. Variants of the complex 17q21 region affecting *GSDMB* and *ORMDL3* were previously found to be determinant for childhood asthma susceptibility and development of Rhinovirus-induced wheezing in preschool age [102,104,105]. Researchers also observed that the effects of the 17q21 genotype on increased predisposition were primarily seen in the subgroup of children with a history of human Rhinovirus (HRV) wheezing illness in early childhood. Only in recent years has the hypothesis of a causative role for 17q21 loci in asthma exacerbation started to be investigated. Although the potential contribution of *ORMDL3* to asthma pathogenesis has been described and it might influence airway hyperreactivity, sphingolipid synthesis, and allergic response [106], there is a lack of studies especially addressing its predictive role on the risk of exacerbations. On the contrary, *GSDMB* showed more promising results. In the Copenhagen Prospective Studies on Asthma in Childhood exacerbation cohort (COPSACexacerbation), composed of children between 2 and 6 years of age with recurrent hospitalizations for asthma attacks, a strong correlation between *GSDMB* and severe exacerbations was described [71]. More recently, a longitudinal study on a cohort of adolescent and adult patients followed for a 3-year period demonstrated that multiple SNPs in *GSDMB* increase its expression and that this correlates with exacerbations [73]. Although the genetic linkage between *GSDMB* and asthma, its biological function in asthma pathogenesis remains unknown. This gene was found to be highly expressed in bronchial epithelial cells and T cells of asthmatic patients [107], and it was associated with a phenotype characterized by diminished lung inflammation and increased airway responsiveness [74]. In vitro studies of *GSDMB* overexpression in human lung epithelial cells showed concomitant upregulation of several genes (like TGF-β1, leukotrienes, chemokines, heat shock proteins), that play a key role in airway hyperresponsiveness [75]. Li et al. also found that the levels of *GSDMB* positively correlate with Major Histocompatibility Complex (MHC) class I molecules, type I IFN and type II IFN pathway genes and Th1 pathway genes, involved in antiviral response [73]. Therefore, individual exacerbation susceptibility might also be influenced by the interaction between viral agents and expression of antiviral pathway genes, modulated by *GSDMB* polymorphisms.

### 4.2. RAD50

DNA Repair Protein RAD50 gene (*RAD50*) is involved in DNA double-strand break repair and its direct role in asthma is not completely clarified. It is located on chromosome 5q31 within the T helper 2 (Th2)-cytokine locus including IL-5, IL-4, IL-13, and it could contribute to tissue-specific Th2 inflammation in asthma and allergic diseases [77,108]. Together with other genes of 5q31 locus, it was identified as a genetic determinant of serum IgE levels [109]. Elevated levels of serum IgE are known to play a key role in allergic asthma, the predominant phenotype during childhood, which also correlates with asthma severity, especially at a young age [110]. Regulation of *RAD50* is complex and implies epigenetic mechanisms, alongside genetic polymorphisms: Michel et al. found that farm environment could influence DNA methylation of asthma- and allergy-related 5q31 genes in early childhood and hypermetilation of *RAD50* could be associated with IgE regulation [111]. Moreover, in children, rs2240032 polymorphisms were demonstrated to impact on *RAD50* expression and correlate with total serum IgE [112]. Li et al. found that SNPs in *RAD50*/*IL13* region on chromosome 5q31.1 were strongly correlated with asthma susceptibility in adults (highest association for rs2244012 in intron 2 of *RAD50*) [78]. Pediatric population has been analyzed in a UK cohort of 370 families with at least two asthmatic children, with no association found between *RAD50* rs2244012 polymorphism and childhood asthma [104]. Meanwhile, Weidinger et al. performed a GWAS in an European cohort of children with atopic dermatitis (AD), showing a significant correlation between *RAD50*/*IL13* locus SNPs and AD, and this association was confirmed for the group with both AD and asthma [113]. After adjustment for race and gender, another *RAD50* SNP (rs2706347) remained significantly related to early-onset allergic asthma in a GWAS conducted on a small cohort of children [77]. The role of *RAD50* rs6871536 in childhood asthma predisposition was also validated in Chinese population [114]. On the contrary, more recent case-control study on 652 Northeastern Han Chinese asthmatic children did not report significant association between two *RAD50* SNPs (rs2244012 and rs6871536) and pediatric asthma risk, questioning the effective role of this gene in this specific subpopulation [115]. The first study to investigate the correlation between RAD50 and asthma exacerbation was conducted on the COPSAC cohort highlighting a genome-wide significant association between *RAD50* rs6871536 polymorphism and early-onset asthma with recurrent and severe exacerbations [71]. Therefore, *RAD50* represents a promising new susceptibility gene for asthma exacerbations, although replication of these results would require more studies, in order to overcome genetic heterogeneity and environmental influences among different populations.

### 4.3. IL-33 and IL1RL1

IL-33 is a member of the IL-1 family. It binds IL-1 receptor–like 1 (IL-1RL1) receptor and plays important role in type-2 inflammation by activating eosinophils, basophils, mast cells, macrophages and group 2 innate lymphoid cells (ILC2s) [116] and IL-33 signaling induces Th2-driven inflammation in allergic asthma. GWAS have reproducibly found that genetic variations in IL-33 and IL1RL1 genes alter IL-33/IL-1RL1 pathway through different molecular changes in gene transcription or protein synthesis, and correlate with increased asthma susceptibility [79]. Beside this, COPSAC study reported that these loci had a larger effect on asthma exacerbation risk rather than on asthma susceptibility in their cohort, highlighting a genetic overlap with significant effects on exacerbation likelihood [71].

### 4.4. IL4RA

IL-4 has a pivotal role in asthma Th2-mediated inflammation and its functions are mediated by the interaction with two receptors: IL-4R type I (composed of the IL-4Rα and the common cytokine receptor γc-chains) and type II (composed of the IL-4Rα and IL-13Rα1 chains) [117]. A study conducted on two adult cohorts showed that SNPs in *IL4RA* (E375A, Q551R) correlate with asthma exacerbation and these variants were more common in African American population. Moreover, a relation between these polymorphisms, higher mast cells count and IgE binding was highlighted [81]. Massoud et al. proposed as underlying mechanism the conversion of induced regulatory T cells toward Th17 cell inflammation driven by altered IL4R function [117]. Studies on children population reported a significant association of *ILR4A* polymorphisms and asthma susceptibility [118], although a more specific predisposition to asthma exacerbation risk needs to be evaluated in these patients.

### 4.5. FLJ22447

Recent evidence suggested that some asthma-related polymorphisms could be ethnical-specific. A new meta-analysis of GWAS conducted on Latino children and adolescents with severe exacerbations reported a significant association with a novel SNP (rs2253681) in *FLJ22447*, the gene of a long non-coding RNA (lncRNA) previously related to myofibroblasts differentiation and airway remodeling. Moreover, they showed that this SNP increased CpG methylation in *FLJ22447*, which was in turn significantly associated with the expression of KCNJ2 antisense RNA 1 gene (*KCNJ2*-*AS1*) in nasal epithelium, recently involved in young atopic asthma [82,83]. Further research is needed to investigate ethnically different cohorts and assess the temporal association of this SNP with severe exacerbations in childhood population.

### 4.6. FCER2

Low-affinity IgE receptor FcεRII(CD23) exists in two forms: a constitutively expressed form (CD23a) on B cells, and an inducible form (CD23b) present on a variety of cells, including myeloid and lymphoid lines, and intestinal epithelial cells. As a receptor, it downregulates IgE response and their synthesis, and can contribute to B cells functions and antigen presentation [119]. SNPs of this gene, like rs3760687, were found to be correlated with mild-to-moderately increased IgE levels in children [120]. Moreover, previous studies demonstrated that total IgE levels may be augmented by corticosteroid therapy. This controversial hypothesis could be explained by the inhibitory effects of corticosteroid on *FCER2* expression and CD23 synthesis, through the action of molecular mediators like IL-2 and IL-4, with subsequent increase in IgE levels [121,122]. Therefore Tantisira et al. evaluated the association of *FCER2* polymorphisms with IgE levels and presence or absence of severe exacerbations over a 4-year clinical trial in the Childhood Asthma Management Program (CAMP) cohort. They identified three SNPs to be significantly associated with elevated 4-year IgE level, increased severe exacerbations and hospitalization risk in white subjects under ICS therapy, and SNP rs28364072(T2206C) to be correlated with risk of exacerbation in both Caucasian and African American populations, despite ICS use [84]. These results were confirmed in a meta-analysis of three pediatric asthma cohorts and in a more recent systematic review on ICS pharmacogenomics, that reported the most significant association for rs28364072 in *FCER2* and risk of asthma attacks, suggesting that it might be a useful predictor of both steroid refractory pediatric patients and exacerbations [85,86].

### 4.7. ALOX5, LTAH4, LTC4S

Leukotriene pharmacogenomics is helpful to understand how genetic variants could influence montelukast response. Two studies focused on the correlation between genetic predisposition and asthma exacerbation risk during treatment. Lima et al. analyzed SNPs in leukotriene pathway candidate gene in a case-control study among adult asthmatic patients receiving montelukast for 6 months: they identified two SNPs in leukotriene C4 synthase gene (*LTC4S*) (rs730012), leukotriene A4 hydrolase gene (*LTA4H*) (rs2660845), and arachidonate 5-lipoxygenase gene (*ALOX5*) haplotype to be associated with increased exacerbation rate, while mutant alleles (X/X and 5/X) in the *ALOX5* gene promoter to correlate with a 73% reduced exacerbation risk compared to wild-type homozygotes [87]. On the contrary, Telleria et al. found that patients homozygous for the wild-type allele had less frequent asthma attacks compared to patients homozygous for the mutant allele after treatment with montelukast [88]. Therefore, results still remain unconclusive and larger studies are needed in order to clarify the clinical relevance of pharmacogenomics related to these genes [86].

### 4.8. ADRB2

The role of different genetic polymorphisms in human β_2_-adrenergic receptor gene (*ADRB2*) has been associated with respiratory system modifications. In particular, Palmer et al. demonstrated that the arginine-16 variant of the Arg16Gly polymorphism in *ADRB2* predisposes to exacerbations in asthmatic children and young adults, mainly in those with regular salmeterol exposure [123]. Basu et al. performed a cohort study on young asthmatics between 3 and 22 years of age and demonstrated that *ADRB2* correlates with increased risk of exacerbation in patients undergoing daily inhaled short or long-β_2_ agonists therapy, while no risk was highlighted for a lower drug use [90]. A meta-analysis of five previously described populations investigated the role of A allele of rs1042713 (resulting in Gly-to-Arg substitution at the 16 position) and long-acting β-agonist (LABA) exposure in pediatric asthma exacerbations, confirming that use of LABA was associated with increased risk in children carrying one or two A alleles. Given the study limitations, clinical trials are required to determine the impact of stratification of treatment by rs1042713 on asthma exacerbation risk in children with mutant alleles [89].

### 4.9. CTNNA3 and SEMA3D

McGeachie et al. performed a GWAS in three cohorts of patients ranging from paediatric to adult age and identified two regulatory SNPs within cathenin alpha 3 gene (*CTNNA3*) and semaphorin 3D gene (*SEMA3D*) that are associated with asthma exacerbations, defined as the need of a 5-day course of oral corticosteroid treatment. *SEMA3D* expression was found to be significantly increased in bronchoalveolar lavage samples and airway smooth muscle cells of asthmatic patients compared to control subjects, differently from *CTNNA3*. SEMA3D product is a member of the semaphorin class 3 signaling molecules and is implicated in endothelial cell migration, angiogenesis and immune cell recruitment during inflammatory response. Therefore, it may contribute to airway remodeling and increase exacerbation risk [124]. Genetic variants in *SEMA3D* are associated with asthma attacks in children cohorts and this result was replicated in an adult biobank population [91]. *CTNNA3* encodes a protein that belongs to the adherence junction complex in epithelial cells and plays a key role for cellular adherence. In children, polymorphisms in *CTNNA3* correlated with asthma response to therapy, in particular ICS treatment, rather than to asthma genetic susceptibility [92]. Interestingly McGeachie et al. GWAS showed that SNPs in *CTNNA3* are genome-wide significant for association with exacerbation in children, regardless of ICS therapy. In relation to this gene, the result was not reproducible in the adult population considered in the study [91]. The exact contribute of these genes to asthma exacerbations susceptibility needs more studies to explain the effects in a population of children and their reproducibility in adult asthma.

### 4.10. CHIT1, CHI3L1, CHIA

Chitinase are a family of hydroxylases with the ability of cleaving chitin, the main component of the fungal cell walls. Chitinase and chitinase-like proteins (chitotriosidase 1 or CHIT1, chitinase 3-like 1 protein or YKL-40, acid mammalian chitinase or AMCase) are encoded by *CHIT1*, *CHI3L1*, *CHIA* genes respectively. Different studies showed that chitinases and derivatives stimulate innate immune cells and type 1 or type 2 inflammation [125]. Consequently, their potential role in asthmatic pathogenesis was investigated. Bierurbaum et al. reported a possible association between *CHIA* polymorphism, coding for AMCase, and paediatric asthma development [126]. Ober et al. demonstrated that a SNP in *CHI3L1* promoter correlated with bronchial hyperresponsiveness, lung function and asthma susceptibility, by studying different cohorts, including adult populations and a birth cohort [93]. However, Wu et al. did not find any relation between SNPs in *CHIT1*, *CHIA* and *CHI3L1* and asthma in CAMP cohort, including children from 5 to 12-year-old [127]. In an adult case-control study, the serum *CHI3L1* encoded protein, called YKL-40, was measured and its levels were found to be significantly elevated in patients with asthma, especially in the group more prone to exacerbations, after stratification [94]. Guerra et al. studied multiple independent child cohorts and described that epigenetic CpG methylation of *CHI3L1* gene affects circulating YKL-40, associated with asthma. Nevertheless, *CHI3L1* SNPs were not significantly related to asthma and association with exacerbation was not investigated in children [95].

### 4.11. Gene-Environment Interaction

#### 4.11.1. *CDHR3*

Cadherin-related family member 3 (CDHR3) is a transmembrane protein that belongs to the cadherin family and is normally expressed in bronchial epithelium. Cadherin proteins are typically involved in multiple cellular functions, like intercellular adhesion and interaction, epithelial morphogenesis and tissue differentiation. They have different sequences, related to their particular affinities and tissue-specific functions [128]. Bønnelykke et al. performed a GWAS in COPSAC cohort and found that this phenotype was associated with a nonsynonymous coding SNP in *CDHR3* locus, in which insertion of allele A resulted in aminoacidic change at position 529, converting cysteine to tyrosine (rs6967330; p.Cys529Tyr). CDHR3 protein has six extracellular domains and this aminoacidic alteration is located at the interface between two different cysteine residues (Cys592 and Cys566), expected to form a disulfide bridge. Consequently, it interferes with protein folding and stability [71]. Bochkov et al. showed that human rhinovirus-C (HRV-C) uses cadherin-related family member 3 (CDHR3) for virus binding and replication in susceptible cells. Moreover, they demonstrated that, compared with wild-type *CDHR3*, cells transfected with the rs6967330 (p.Cys529Tyr) variant had a more significant CDHR3 protein surface expression and a 10-fold increase in HRV-C binding and progeny growth in vitro [96]. As HRV-C is known to be the principal trigger of exacerbations in asthmatic children, causing more severe attacks and higher need of hospitalization than other HRV groups [129], it is plausible that genetic polymorphisms affecting its cellular receptor and increasing its membrane expression underlie the higher susceptibility in this subset of young patients. In 2019 Everman et al. tried to better understand the role of *CDHR3* in virus-induced asthma exacerbations by studying human airway epithelial cells (AECs) from donors. They discovered that *CDHR3* expression is restricted to ciliated cells and recognized that HRV-C has exclusive tropism for these cells in the human lung. In addition, by creating CDHR3 knockout AECs, they demonstrated that HRV-C infection was reduced by 80% in these cells, strongly supporting the role of CDHR3 as a receptor for HRV-C cellular infection. The rs6967330 variant in *CDHR3* gene was confirmed to be a risk factor for severe childhood asthma exacerbations, probably by increasing HRV-C infection (7.5-fold) and protein surface expression [97]. Altered CDHR3 impairs airway epithelium integrity and plausibly increases the susceptibility to viral infection, pollutants and other irritants, promoting recurrent exacerbations [71].

#### 4.11.2. Other Gene-Environment Interactions

The transforming growth factor-β group consists of approximately 30 cytokines. TGFβ1, a pleiotropic fibrogenic and immunomodulatory factor, is involved in asthma, both in the airway inflammatory response and in the fibrotic remodeling [130]. Sharma et al. performed the first study of association between variants of TGFβ1 and disease exacerbation in subjects with asthma. They examined two cohorts of children and showed that T allele of a coding polymorphism in *TGFB1* (rs1982073) was inversely associated with exacerbations. Moreover, dust mite exposure significantly modified the effect of the A allele of the promoter SNP rs2241712 on asthma exacerbations in both cohorts [98]. IL-9 is a cytokine produced by Th2 cells and mast cells and it was found to be involved in gene-environment interaction in asthma. SNPs rs11741137/rs2069885 in IL-9 gene of patients concomitantly exposed to high levels of house dust mite allergen were correlated to the highest frequency of severe exacerbations. This effect seemed to decline when the exposure to the allergen was low [76]. Class-I MHC-restricted T cell associated molecule (CRTAM) is a surface marker especially expressed on activated class-I MHC-restricted T cells, including CD8 and NK human cells. It can bind antigen-presenting cells and promote NK cytotoxicity or interleukin secretion by CD8 lymphocytes [131]. Du et al. conducted a GWAS to investigate the association between vitamin D levels and risk of asthma attacks on a cohort of children, by analyzing different genetic polymorphisms. They identified 3 common variants *CRTAM* to be associated with an increased exacerbation rate in case of concomitant low circulating vitamin D levels (defined as ≤30 ng/mL), while high vitamin levels appeared to be protective against asthma attacks for the same variants. Moreover, they discovered that the concurrent presence of rs2272094 variant and low vitamin D levels reduced *CRTAM* expression, which may in turn be a risk factor for increased asthma exacerbations. Thus, they suggested that maintaining adequate levels of vitamin D would be appropriate for high-risk asthmatic patients [99]. This result highlighted a new important gene-environment interaction. It was supposed that *CRTAM* could influence immunity against viral infections [100], although the biological mechanisms behind the role of vitamin D in this context are still unclarified.

### 4.12. Transcriptomics

Recent studies focused on the role of microRNAs in asthma pathogenesis, showing promising results. MicroRNAs (miRs) are small noncoding RNAs that can be measured in peripheral blood and can regulate gene expression at the post-transcriptional level. There is growing interest on the future perspective of using microRNAs as outcome biomarkers of many diseases and, in particular, as exacerbation predictors in asthma. Midyat et al. analyzed 100 asthmatic children compared to equal age-matched controls and demonstrated that expression of 10 microRNAs was higher in patients with more severe asthma and exacerbation symptoms [132].

In Tian et al. case-control study 100 children with asthma attacks were found to have lower levels of miR-1 and interferon-*γ* (IFN-*γ*) and higher levels of IL-4, IL-5, IL-8, and tumor necrosis factor-alpha (TNF-*α*), in the peripheral blood, with wider changes observed in those with severe asthma. The downregulation of miR-1 showed great accuracy as a potential index for evaluating acute-stage asthma and could correlate with the imbalance in T-helper function observed during asthma attacks [133]. In 2018 Kho et al. were the first to investigate the role of microRNAs in predicting asthma exacerbations, by examining a cohort of 153 asthmatic children prior to treatment with inhaled corticosteroid (ICS). They observed that the combination of three microRNAs (miR-146b, miR-206 and miR-720) involved in NF-kβ (nuclear factor kappa-light-chain-enhancer of activated B cells) and glycogen synthase kinase-3 (GSK3)/AKT pathways was more accurate than an established clinical exacerbation score in predicting future asthmatic exacerbations. They also showed that if microRNAs and clinical score were associated, their predictive power increased [134]. Therefore, beside their potential role as biomarkers of asthma exacerbations, microRNAs could also significantly contribute to the prediction of exacerbations.

Recently, long non-coding RNAs showed promising roles in different diseases and their contribution to asthma started to be investigated. A systematic transcriptome analysis on peripheral whole blood of a small group of asthmatic children reported that many of the key non coding RNA molecules found were related to asthma susceptibility genes, particularly in those involved in immune, inflammatory and apoptosis processes [135]. In the past, LncRNA nuclear-enriched abundant transcript 1 (lncRNA NEAT1) showed a significant role in adult asthma. It derives from NEAT1 and it interacts with microRNA (miR-124) through a competitive endogenous RNA regulation (ceRNA). miR-124 contributes to anti-inflammatory phenotype in asthmatic lung macrophages, whereas lncRNA NEAT1 plays an important role in inflammasome activation and cytokine regulation and is negatively associated with miR-124 [136]. Li et al. studied these molecules in asthmatic adult population compared to healthy controls and reported lncRNA NEAT1 expression to be of good predictive value for asthma exacerbation risk and severity. Moreover, it was found that miR-24 was inversely associated with asthma attacks and inflammation, so it is likely that lncRNA NEAT1 might contribute to asthma pathogenesis via interaction with miR-124 [137]. Thereby, lncRNAs seem to be potential new biomarkers, although more studies are needed to validate their role in asthma exacerbation risk, especially in children cohorts, not yet investigated.

## 5. Conclusions

Asthma exacerbations represent a major global burden in asthmatic patients, especially in young children. These events have a large impact on quality of life of patients and caregivers, limiting social and physical activities [138,139]. Recurrent exacerbations induce airway remodeling and lung function impairment, worsening asthma severity and increasing the risk of potential life-threatening events. These are relevant issues in both severe and mild-moderate asthma. Notably, mild asthma is often underdiagnosed and undertreated despite the consistent risk of near-fatal events [12,14]. Prevention of asthma attacks becomes crucial in the management of all patients. This goal requires a better characterization of the exacerbation-prone phenotype.

Biomarkers and genomics may be helpful tools. In particular, easy-to-perform tests are necessary in a population of children, in which sample collection (e.g., sputum) is often complicated. B-Eos and FeNO are the most studied markers and many works have reported their predictivity for exacerbations. Some studies did not confirm their role, probably due to the variability of their levels. This suggests the need of serial measurements and the use of composite markers. Interest has increased in the use of more feasible urinary and salivary samples, as well as exhaled breath, on which stronger efforts should focus. IL-6 and microRNA represent further promising markers. Research on genetic predisposition, through genome-wide association studies or specific gene findings, highlighted an increasing number of genetic polymorphisms involved. Many works reported genetic overlap between previously described asthma susceptibility genes and exacerbation-associated loci, like 17q21 region. Significant associations were also found for gene-environment interaction, showing genome-wide differential gene expression as a result of extrinsic factors influence. In addition, genes like *CDHR3* have been found to specifically correlate with exacerbations.

Given the heterogeneity of the populations considered, further studies are needed to improve and validate the role of genomic in clinical settings. In conclusion, these findings support the growing importance of an individual-shaped management, that considers not only the severity degree of asthma but also the risk of recurrent exacerbations. Based on the results of this review, a practical approach that we may suggest should include the use of easy to obtain laboratory biomarkers, in order to stratify individual risk at both diagnosis and follow-up. The majority of evidences favors the use of B-Eos and FeNO as exacerbation predictors: according to risk quantification data reported, cut-off points of 300 cells/μL for B-Eos, and of 48 ppb for FeNO may be considered indicative of high exacerbation risk, despite the current lack of a single validated threshold. Further studies on non-invasive biomarkers may improve the feasibility and accuracy of stratification, maybe through the use of composite markers. In childhood, annual clinical and laboratory assessment should be considered, shortening follow-up timing in case of high-risk quantification.

In addition, exacerbation risk evaluation may be a useful tool in guiding therapeutic strategy: the high-risk stratification should encourage the choice of a controller therapy rather than reliever-only approach in mild-asthma patients. Therefore, follow-up planning and therapy optimization should aim to avoid exacerbations, preventing airway remodeling and decreasing asthma-related morbidity and mortality.

## Figures and Tables

**Table 1:** Summary of studies investigating the role of biomarkers in asthma exacerbations.

Biomarkers	Exacerbation Prediction				Other Purposed Uses in Literature
	Population Age	Quantitative Cut-Offs/ Qualitative Panels	OR/RR	[Ref.]	
**B-Eos**	5–12 years	Higher log 10 eosinophil count		[18]	- Disease phenotyping - Evaluating asthma severity - Monitoring of asthma control - Prediction of treatment response
	6–17 years	≥300/mmc	OR 1.35	[19]	
	≥6 years	≥300/mmc	OR 1.60 with Eos 300-500/mmc; OR 2.19 with Eos > 500/mmc	[20]	
	5–11 years	≥300/mmc	OR 1.52	[21]	
	≥12 years	≥400/mmc	OR 1.48	[22]	
	5–11 years ≥12 years	≥150/mmc ≥300/mmc	OR 2.39 OR 2.04	[23]	
	>12 years	≥450/mmc	OR 1.33–1.84	[24]	
**FeNO**	6–18 years	>49 ppb		[25]	- Disease phenotyping - Evaluating asthma severity - Monitoring of asthma control
	6–18 years	>22.9 ppb		[26]	
	12–56 years	≥48 ppb	RR 2.4; compared with FeNO < 20ppb	[27]	
	6-12 years >12 years	20–35 ppb (intermediate), > 35 ppb (high) 25–50 ppb (intermediate), >50 ppb (high)	OR 1.44 with intermediate values, OR 2.32 with high values	[20]	
**VOCs**	6–16 years	7 VOCs profile		[28]	- Early asthma diagnosis - Disease phenotyping
	6–18 years	15 VOCs profile		[29]	
	6–17 years	7 VOCs profile		[30]	
**EBC** 8-isoprostane	6–18 years	Higher: mean value 114 pg/mL in ≥4 exacerbation/y vs. 52 pg/mL in 1–3 exacerbation/y)		[31]	- Disease phenotyping - Evaluating asthma severity - Monitoring of asthma control
IL5	6–16 years			[32]	
**IL6**	6–17 years	(Risk increased of 24% for each quartile increase in baseline IL-6 level (interquartile range, 0.39–1.65 pg/mL))		[33]	- Evaluating asthma severity
	≥12 years	≥3.1 pg/mL	OR 1.24	[34]	
**Urinary** **metabolites** Urinary Bromotyrosine	6–21 years	(higher: range 0.02–0.68 with increased risk vs. 0.00–0.18 with lower risk)	OR 4	[35]	- Disease phenotyping - Monitoring of asthma control - Prediction of treatment response - Evaluating asthma severity - Predicting exacerbations - Prediction of treatment response - Monitoring of asthma control
Urinary leukotriene E4	6–15 years	>106 pg/mg		[36]	
Isoprostane	>10 years	(higher)		[37]	
**Salivary** **metabolites**	7–16 years	-		[38]	- Asthma phenotyping - Monitoring of asthma control

OR = odds ratio; RR = relative risk; B-Eos = blood eosinophils; FeNO = fractional exhaled nitric oxide; VOCs = volatile organic compounds; EBC = exhaled breath condensate; IL = interleukin.

**Table 2:** Summary of genetic variants associated with asthma exacerbations.

Gene	Gene Product	Association	References
*GSDMB*	Gasdermin B	Asthma exacerbation GSDMB expression Asthma susceptibility Asthma severity Antiviral pathways	[71,73,74,75,76]
*RAD50*	DNA repair enzyme	Asthma exacerbation Asthma susceptibility Th2 inflammation	[71,77,78]
*IL-33, IL1RL1*	IL-33/ILRL1 (ST2) pathway	Asthma exacerbation Eosinophilic asthma	[71,79,80]
*IL4RA*	Interleukin-4 receptor	Asthma exacerbation Elevated mast cells, IgE levels	[81]
*FLJ22447*	Long non-coding RNA	Severe asthma exacerbation	[82,83]
*FCER2*	Low-affinity IgE receptor FcεRII(CD23)	Asthma exacerbation Poor ICS response	[84,85,86]
*ALOX5, LTAH4, LTC4S*	Leukotrienes pathway	Asthma exacerbation in montelukast-treated	[86,87,88]
*ADRB2*	Adrenergic β_2_-receptor	Asthma exacerbation in SABA or LABA-treated	[89,90]
*CTNNA3,* *SEMA3D*	Cathenin alpha 3 Semaphorin class 3 D	Asthma exacerbation Airway remodelling	[91,92]
*CHIT1, CHI3L1, CHIA*	CHIT1, YKL-40, AMCase	Asthma exacerbation in adults Asthma susceptibility	[93,94,95]
*CDHR3*	Cadherin-related family member 3	Asthma exacerbation CDHR3 expression HRV-C infection	[71,96,97]
*TGF-β*	Transforming growth factor-β	Asthma exacerbation modified by HDM exposure Airway hyperresponsiveness	[98]
*IL-9*	Interleukine-9	Asthma exacerbation Associated with HDM exposure	[76]
*CRTAM*	Class-I MHC-restricted T cell associated molecule	Asthma exacerbation in low vitamin D levels	[99,100]

SABA = short-acting beta agonists; LABA = long-acting beta agonists; ICS = inhaled corticosteroids; HRV = human rhinovirus; HDM = house dust mite.

## Supplementary Materials

The following are available online at https://www.mdpi.com/article/10.3390/ijms22094651/s1, Figure S1: Review phases flowchart.
