# Supplementary figures and images for: The Predictive Role of Biomarkers and Genetics in Childhood Asthma Exacerbations
Source: Int J Mol Sci. 2021 Apr 28;22(9):4651. doi: 10.3390/ijms22094651 (PMC8124320; doi:10.3390/ijms22094651)

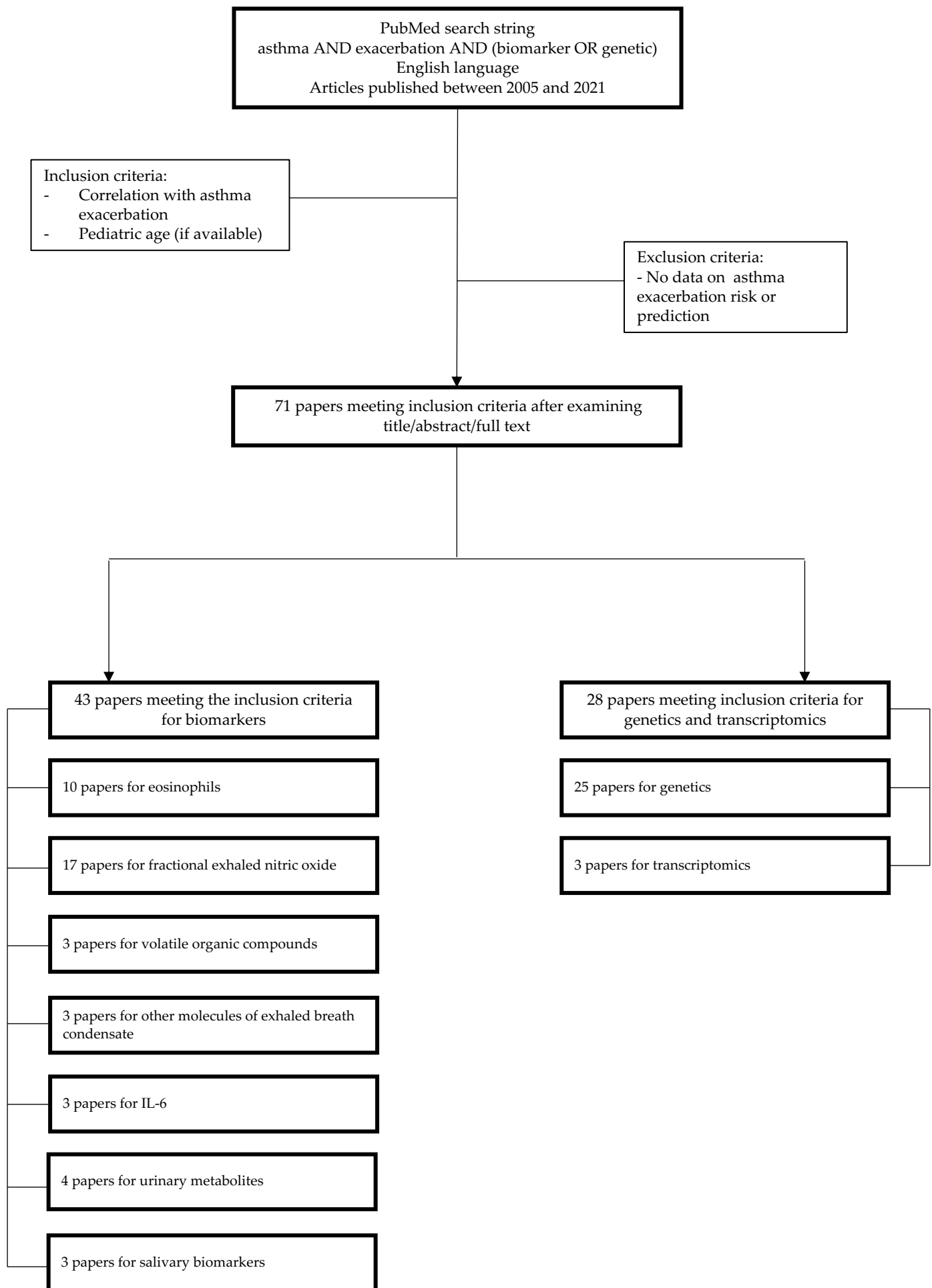

**Figure S1-** Review phases flowchart.

Supplement: Supplementary file 1 [file ijms-22-04651-s001.zip › ijms-1160828-supplementary.pdf]
